# Multi-omics integration identifies ARID1B linking cuproptosis-immune crosstalk with atherosclerotic plaque progression

**DOI:** 10.3389/fgene.2026.1795872

**Published:** 2026-05-18

**Authors:** Xinyan Hu, Kunpeng Wei, Pengfei Pang, Wuguang Chang, Lin Huang

**Affiliations:** 1 Department of Interventional Medicine, The Fifth Affiliated Hospital of Sun Yat-sen University, Zhuhai, China; 2 The Fifth Affiliated Hospital of Sun Yat-sen University, Zhuhai, China

**Keywords:** ARID1B, atherosclerosis, cuproptosis, immune infiltration, machine learning

## Abstract

**Background:**

Atherosclerosis is a long-term inflammatory disorder of the arterial wall, characterized by lipid deposition and infiltration of immune cells. Recent studies suggest that cuproptosis may participate in the progression of atherosclerosis.

**Methods:**

Multiple scRNA-seq and bulk RNA-seq datasets were integrated to systematically characterize cuproptosis patterns in atherosclerosis. Differential expression analysis based on cuproptosis scores identified cuproptosis-modulating genes, which were further refined using least absolute shrinkage and selection operator, decision tree, and XGBoost algorithms. CellChat analysis explored the effect of ARID1B expression on intercellular communication, while immune infiltration patterns were assessed via single-sample gene set enrichment analysis. ApoE^−/−^ mice served as the *in vivo* model of atherosclerosis to confirmed relationships among cuproptosis, ARID1B expression, and immune infiltration in plaques.

**Results:**

Cuproptosis activity was significantly elevated in atherosclerotic tissues compared with normal arteries, with macrophages exhibiting the highest cuproptosis scores. Distinct expression profiles of cuproptosis-related genes, together with divergent immune infiltration landscapes, were observed between the high and low cuproptosis score atherosclerosis groups. ARID1B was identified as a gene discriminating cuproptosis status in atherosclerosis pathology, with significantly reduced expression in the disease group. Functional enrichment analyses suggested that ARID1B-associated networks were mainly involved in immune cell adhesion, activation, and differentiation. *In vivo* validation further confirmed that ARID1B expression was significantly reduced in the atherosclerosis group, which was associated with prominent T cell and macrophage infiltration.

**Conclusion:**

Cuproptosis-related gene ARID1B may reshape immune infiltration patterns in atherosclerosis, positioning it as a promising therapeutic target.

## 1 Introduction

Atherosclerosis is a vascular disease characterized by dysregulated lipid metabolism and chronic inflammation, ultimately leading to luminal stenosis or acute thrombotic occlusion following plaque rupture or erosion (Bentzon et al., 2014). As the pathological basis of many cardiovascular diseases, it remains a major cause of death worldwide (Herrington et al., 2016). Although lipid-lowering therapies have improved clinical outcomes, substantial residual risk persists, and disease progression is not fully controlled (Libby, 2021). During disease progression, endothelial dysfunction and low-density lipoprotein cholesterol deposition promote inflammatory cell recruitment. As macrophages, T cells, and other immune cells accumulate within plaques, programmed cell death (PCD) increases and a necrotic core forms, ultimately driving plaque progression toward vulnerability (De Meyer et al., 2024). However, the specific roles and regulatory mechanisms of different forms of PCD in atherosclerosis remain to be fully elucidated.

Cuproptosis is a recently identified form of cell death triggered by abnormal intracellular copper accumulation (Yang et al., 2024c). Its core mechanism involves copper binding to lipoylated mitochondrial proteins, thereby inducing lipoylated protein aggregation, destabilization of iron-sulfur cluster proteins, and proteotoxic stress, ultimately leading to cell death. FDX1 and LIAS, members of iron-sulfur cluster proteins, are upstream regulators of protein lipoylation, thereby establishing their role as key factors in cuproptosis. The oligomerization of lipoylated DLAT, an essential component of the pyruvate dehydrogenase (PDH) complex, results in a toxic gain of the citrate cycle (TCA cycle) function (Tsvetkov et al., 2022). As an essential trace element, copper plays a critical role in fundamental biological processes, including immune homeostasis and antioxidant defense (Arredondo and Núñez, 2005; Zhang et al., 2023). However, increasing industrialization and mining activities have markedly elevated environmental copper release, making it a persistent pollutant with important public health implications (Li et al., 2021). Recent studies have shown that, beyond tumors and inherited disorders of copper metabolism, cuproptosis-related molecules are also involved in cardiovascular diseases such as atherosclerosis and heart failure, suggesting that cuproptosis may represent a novel mechanism and potential therapeutic target in atherosclerosis (Chen X. et al., 2023; Eshak et al., 2018; Lin et al., 2024; Yang et al., 2022; Yang S. et al., 2024; Zou et al., 2025). Quantitative analysis of vascular tissues confirmed significantly elevated copper concentrations in atherosclerotic plaques compared to healthy controls (Stadler et al., 2004). Elevated serum copper concentration has been established as an independent risk factor for cardiovascular diseases (Yang S. et al., 2024). In addition, the high-cuproptosis subtype exhibits more prominent immune infiltration in cerebral cavernous malformations, particularly enhanced macrophage infiltration (Chen et al., 2024a), indicating that cuproptosis may drives vascular disease progression through remodeling the immune microenvironment. However, the specific characteristics of the interaction between cuproptosis and immune infiltration, as well as the regulatory molecules involved in atherosclerosis, remain to be elucidated.

In this study, multiple scRNA-seq and bulk RNA-seq datasets were integrated to systematically characterize cuproptosis-associated immune remodeling in atherosclerosis. Cuproptosis activity was significantly elevated in atherosclerotic tissues and was accompanied by distinct immune infiltration patterns. Machine learning analyses combined with *in vivo* validation identified ARID1B as a downregulated cuproptosis-associated gene closely linked to T cell and macrophage infiltration, suggesting that it may serve as a molecular link between cuproptosis and immune microenvironment remodeling during plaque progression.

## 2 Materials and methods

### 2.1 Data collection

Four scRNA-seq datasets were integrated from the Gene Expression Omnibus (GEO) (GSE159677,GSE216860, GSE196943, and GSE253903), comprising a total of 27 atherosclerosis samples and 9 normal blood vessels. Bulk RNA-seq data were obtained from GSE100927 (containing 69 atherosclerosis samples and 35 normal blood vessels) and GSE43292 (32 atherosclerosis samples). 18 established cuproptosis-related genes were identified through published literature and validated as critically involved in cuproptosis molecular mechanisms or pathway regulation (Chen W. et al., 2023; Liu et al., 2023; Tang et al., 2024; Tsvetkov et al., 2022) (Supplementary Table S1). Among these, genes encoding lipoic acid pathway components or lipoylation targets were specifically confirmed as direct markers of cuproptosis through genome-wide CRISPR/Cas9 loss-of-function screens performed by Tsvetkov et al. Copper transporter-encoding genes are proved significant association with cuproptosis activation (Tsvetkov et al., 2022).

### 2.2 Cuproptosis scoring

For scRNA-seq data, the cuproptosis scores were calculated across all atherosclerosis samples through application of the Gene Set Variation Analysis (GSVA) (Hänzelmann et al., 2013). The expression matrix used as input was log normalized counts generated by Seurat’s NormalizeData function. The analysis was performed with the following parameters: method = “gsva” (the classic GSVA algorithm), kcdf = “Gaussian” (appropriate for log normalized single cell expression data), min.sz = 1, max.sz = Inf (to include the full gene set), mx.diff = TRUE (to calculate enrichment scores as the maximum deviation from the null distribution). For each cell, GSVA yields a continuous enrichment score representing the relative activity of the cuproptosis pathway, with higher scores indicating stronger pathway activation. For bulk RNA-seq data, the ssGSEA algorithm was employed to compare cuproptosis levels across atherosclerosis samples. Samples were categorized as cuproptosis high or low based on median values.

### 2.3 Processing and analysis of scRNA-seq data

After merging all samples, the following steps were performed sequentially: “NormalizeData”, “FindVariableFeatures”, “ScaleData”, and “RunPCA”. Quality control was performed using the Seurat package. Cells were filtered based on the percentage of mitochondrial genes (percent.mt), the number of detected genes (nFeature_RNA), and the total number of UMI counts (nCount_RNA). Only cells with percent.mt < 15%, nFeature_RNA between 200 and 6,000, and nCount_RNA between 200 and 20,000 were retained for downstream analysis. Subsequently, the “harmony” R package was used to remove batch effects (Korsunsky et al., 2019). All markers used for cell identification are recognized or documented in previous researches: myeloid cells (CD14, CD68, and CD163), T cells (CD3D, CD3E), fibroblasts (ACTA2, COL1A1, COL1A2, and RGS5), B/plasma (CD79A, MS4A1, and JCHAIN), endothelial cells (VWF and PECAM1), epithelial cells (EPCAM and CLDN4), and mast cells (TPSAB1 and TPSB2).

### 2.4 Identification of differentially expressed genes

As previously described (Yang et al., 2024b), the “FindMarkers” function in the ‘Seurat’ R package was used to identify differentially expressed genes for scRNA-seq data in samples with high and low cuproptosis scores. For bulk RNA-seq data, the “limma” R package was employed to analyze atherosclerosis samples with high and low cuproptosis levels, with adjusted p-values <0.05 being included in subsequent analyses.

### 2.5 Machine learning algorithms for identifying key genes

In this study, we employed and compared three distinct machine learning algorithms: Least absolute shrinkage and selection operator (LASSO) regression, Decision Tree, and Extreme Gradient Boosting (XGBoost). The LASSO model was applied to perform variable selection and enhance model interpretability through its L1 regularization penalty, with the optimal regularization strength (λ) determined via cross-validation. The Decision Tree algorithm was utilized to capture non-linear relationships and interactions within the data; parameters such as maximum depth were tuned to prevent overfitting. For superior predictive performance, the XGBoost model, an advanced ensemble method based on gradient boosting, was implemented. It combines multiple weak learners sequentially and incorporates L1 and L2 regularization to control model complexity. Hyperparameters for all models were rigorously optimized using cross-validated search techniques to ensure robustness and generalizability.

### 2.6 Single-cell metabolic pathway activity

Metabolic pathway scores were assigned to cells in all atherosclerosis samples using the “scMetabolism” R package (Wu et al., 2022). Metabolic pathways were sourced from the Kyoto Encyclopedia of Genes and Genomes (KEGG) and Reactome databases.

### 2.7 Differences in cellular communication in the microenvironment at different ARID1B levels

Based on the “CellChat” R package (Jin et al., 2021), we performed cellular communication analysis on ARID1B-high and ARID1B-low in atherosclerosis, and compared differences in key functional pathways between the 2 cell groups.

### 2.8 Analysis of immune infiltration

Employing the “GSVA” R package (Hänzelmann et al., 2013), we conducted an analysis of immune cell infiltration using two bulk RNA-seq datasets, comparing the relative levels of 28 distinct immune cell types. Sankey diagram was generated using “networkD3” R package.

### 2.9 PPI network construction and GO enrichment analysis

A protein-protein interaction (PPI) network was generated for ARID1B along with its 20 most closely interacting genes using the STRING database (https://cn.string-db.org), and Gene Ontology (GO) enrichment analysis was performed on these genes with the “clusterProfiler” R package (Yu et al., 2012). Metabolic pathway activity was quantified using the scMetabolism R package. The score reflects coordinated expression of all genes within a defined metabolic gene set. Higher scores indicate stronger pathway activity.

### 2.10 Animals

All experimental protocols involving animals were approved and reviewed by the Animal Care Committee of The Fifth Affiliated Hospital of Sun Yat-sen University (Approval No. 00344) and conducted in strict accordance with the National Institutes of Health Guide for the Care and Use of Laboratory Animals. Five-week-old male ApoE^−/−^ mice with C57BL/6J genetic background were obtained from Vital River Laboratory Animal Technology Co., Ltd. (Beijing, China). Upon arrival, the five-week-old ApoE^−/−^ mice were randomly assigned to either regular chow diets or high-fat diet (HFD) feeding regimens. Atherosclerotic lesions were induced by feeding the animals an HFD (Cat#D12108C, 40% kcal fat, 1.25% cholesterol, Beijing Keao Xieli Feed Co., Ltd., China) for 12 weeks. Regular chow diets (Cat#1025, Beijing HFK Bioscience Co., Ltd., China) were applied to control groups for 12 weeks. To maintain consistency in copper exposure across experimental groups, the atherogenic diet was specifically formulated to match the copper content of the regular chow diet. These mice were maintained under standardized pathogen-free conditions (24 °C ± 2 °C, 40% ± 5% humidity, and a 12-h light/dark cycle) to minimize environmental confounding factors. After the 12-week treatment period, mice were humanely euthanized with intraperitoneal injection of sodium pentobarbital anesthesia (250 mg/kg). Perfusion was performed with phosphate-buffered saline (PBS, pH 7.4) for 5 min via left ventricular puncture to ensure complete blood clearance. Tissue samples were preserved in 4% paraformaldehyde for further analyses.

### 2.11 Cell culture and gene knockdown

THP-1 monocytes (Cat#SCSP-567, National Collection of Authenticated Cell Cultures, China) were cultured in RPMI-1640 medium (Cat#11875093, Gibco, United States) supplemented with 10% fetal bovine serum (FBS). Cell line authentication was performed by short tandem repeat (STR) profiling, with routine *mycoplasma* testing confirming absence of contamination. THP-1 monocytes were differentiated into macrophages by treatment with 50 ng/mL phorbol 12-myristate 13-acetate (PMA, Cat#P8139, Sigma Aldrich, United States). After 48h incubation, differentiated macrophages were at 70%–80% confluency. ARID1B-targeting siRNA (Cat#RY26019026, IGE biotechnology Co., Ltd, China) and scrambled negative control siRNA were transfected into THP-1 macrophages using Lipofectamine RNAiMAX (Cat#13778075, Invitrogen, United States) following the manufacturer’s instructions. Cells were maintained in transfection mix for 6h at 37 °C, then replaced with complete medium supplemented with 1 μM CuCl_2_. The sequence for si-ARID1B was: 5′-GGA​GAA​AUU​CUA​UGC​UAC​ATT-3’; 5′-GCU​GCG​AAC​UCA​GCA​CAA​ATT-3’.

### 2.12 Oil red O (ORO) staining

Aortic root tissues were embedded in optimal cutting temperature (OCT) compound and serially sectioned at a thickness of 10 μm. These sections underwent fixation in 4% paraformaldehyde for 12 min, followed by exposure to 60% isopropanol for 5 min. Subsequently, sections were incubated in 0.5% ORO solution in isopropanol for 30 min. After staining, sections were cleansed with distilled water and nuclei were highlighted through hematoxylin counterstaining for 1 min. The lesion area was quantified by calculating the percentage of ORO-positive area relative to the total aortic root area.

### 2.13 Western blotting

Total protein was extracted in RIPA lysis buffer. Protein concentrations were determined to ensure loading accuracy via a bicinchoninic acid (BCA) assay kit (Beyotime, P0012). Lysates were resolved electrophoretically on 10% or 12.5% sodium dodecyl sulfate-polyacrylamide gel electrophoresis (SDS-PAGE) gels (Epizyme, PG212/PG213) under reducing (20 mM DTT) or non-reducing conditions. Separated proteins were transferred from the gel onto polyvinylidene difluoride (PVDF) membranes (Millipore, 3010040001). For immunoblotting, membranes were incubated with the following validated primary antibodies: DLAT (Cat#MA5-24863, Invitrogen), Ferredoxin 1 (FDX1, Cat#ab108257, Abcam), β-actin (Cat#ZB15001-HRP-100, Servicebio), LIAS (Cat#11577-1-AP, Proteintech). Band intensities were quantified using ImageJ. IntDen values were background-corrected and expressed relative to β-actin.

### 2.14 Serum copper measurement

Serum copper concentrations were measured colorimetrically using a Copper (Cu) Colorimetric Assay Kit (Cat#E-BC-K300-M, Elabscience, Wuhan, China). The assay was performed in accordance with the manufacturer’s guidelines, utilizing 15 μL serum. Serum aliquots were diluted to recommended volumes with assay buffer. Sample absorbance values were interpolated against a standard curve generated from known concentrations to calculate final serum copper levels.

### 2.15 Immunohistochemical staining

For immunohistochemical evaluation, tissue sections were fixed with 4% paraformaldehyde, exposed to 3% hydrogen peroxide, and incubated with goat serum blocking solution (Cat#AR0009, Wuhan Boster Biological Technology, Wuhan, China). The primary antibody against LIAS (Cat#11577-1-AP, Proteintech), diluted at 1:500, was applied and incubated overnight at 4 °C. After incubating with biotinylated secondary antibodies, antigen detection was accomplished using 3,3′-diaminobenzidine (DAB) as the chromogenic substrate. Nuclear counterstaining was subsequently performed using hematoxylin. These sections were then evaluated by light microscopy to determine LIAS protein expression patterns in atherosclerotic lesions. The H-score was analyzed using CaseViewer and QuantCenter (v.2.2).

### 2.16 Immunofluorescence staining

After fixation, frozen aortic root sections were permeabilized with 0.3% Triton X-100 for 10 min and subsequently blocked using goat serum for 1 h. Sections were treated overnight at 4 °C with primary antibodies directed against CD68 (Cat#65187-1-Ig, Proteintech, diluted 1:200), ARID1B (Cat#bs-12520R, Bioss, diluted 1:100), or CD3 (Cat#14-0032-81, Invitrogen, diluted 1:200). After thorough washing, Alexa Fluor-conjugated secondary antibodies were administered for 1 h at room temperature under light-protected conditions. The sections were visualized by a fluorescence microscope after mounting using Fluoroshield mounting medium containing 4′,6-diamidino-2-phenylindole (DAPI, Cat#ab104139, Abcam, Cambridge, United Kingdom). Mean fluorescence intensity and area were analyzed using CaseViewer (v.2.2) or ImageJ (v.1.8.0).

### 2.17 Statistical analysis

Distribution normality was examined through the Shapiro–Wilk test, and variance homogeneity was verified using Levene’s test. Student’s unpaired t-test or Mann-Whitney U test was used for the comparison between the two groups. One-way ANOVA test was used to compare differences between multiple experimental groups. Correlation analyses were conducted using Pearson’s correlation coefficient for normally distributed continuous variables and Spearman’s rank correlation coefficient for non-normally distributed data. A minimum of three independent experimental replicates were performed, with “n” representing biological replicates. Results are reported as the mean ± standard deviation (SD). All statistical analyses were performed using GraphPad Prism software (v.8.0.1), with statistical significance set at *P* < 0.05.

## 3 Results

### 3.1 Characterization of atherosclerosis at the single-cell level

**FIGURE 1:**
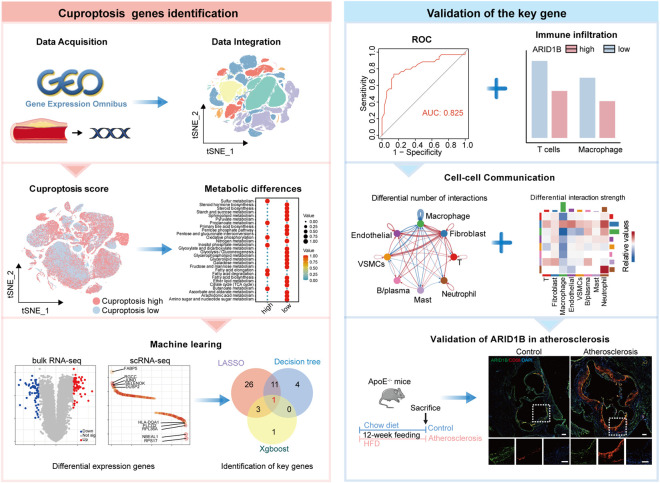
Flowchart in this study.

**FIGURE 2:**
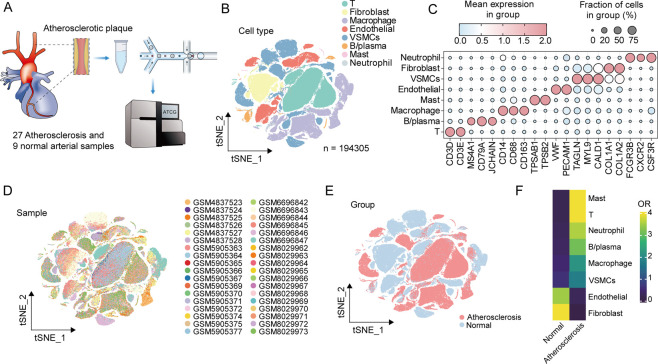
The single-cell landscape of atherosclerosis. **(A)** Data collection process. **(B)** TSNE plot of all cells. **(C)** Identify different cell types based on known markers. **(D)** Distribution and cluster abundance analysis of different samples in all cells. **(E)** Distribution of different cells in atherosclerosis and normal vessels. **(F)** Distribution preferences of different cells in atherosclerotic and normal vessels. OR: odds ratios.

The research workflow was illustrated in Figure 1. We integrated scRNA-seq data from 36 samples, yielding a total of 194,305 cells after quality control. Based on characteristic markers, we identified major cell types, including T cells, B/plasma cells, macrophage, mast, neutrophil, vascular smooth muscle cells (VSMCs), endothelial cells, and fibroblast cells (Figures 2A–C). Cell distribution across samples indicated batch effects were effectively eliminated (Figure 2D; Supplementary Figure S1A). Distinct cellular distributions were observed between atherosclerosis and normal tissues: immune cells and VSMCs predominantly populated in atherosclerosis group, while endothelial, and fibroblast were enriched in normal vascular tissue (Figures 2E,F). This pattern indicates significant alterations in the immune microenvironment of atherosclerosis patients.

### 3.2 Characteristics of cuproptosis-related genes in atherosclerosis at the single-cell dimension

**FIGURE 3:**
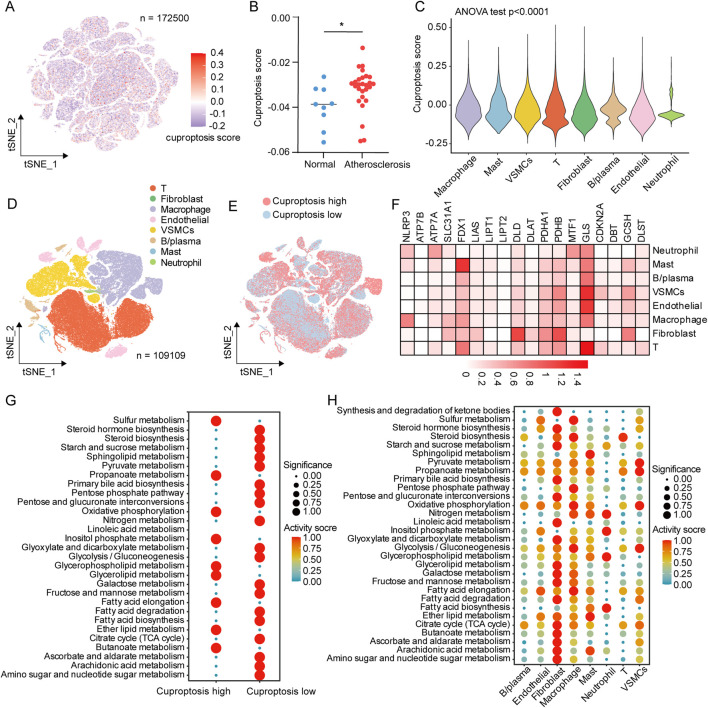
Characteristics of cuproptosis at the single-cell level in atherosclerosis. **(A)** Distribution of cuproptosis scores across all cells. **(B)** Differences in cuproptosis score between atherosclerosis and normal vessels. **(C)** Differences in cuproptosis score among all cell types. **(D)** UMAP dimensionality reduction plot of all atherosclerosis samples after excluding normal vascular tissues. **(E)** Distribution of samples with high and low cuproptosis scores in atherosclerosis group. **(F)** Differential expression of cuproptosis-related genes in different cell types. **(G)** Differences in metabolic pathways in the high and low cuproptosis score group. **(H)** Differences in metabolic pathways in different cell types. *P < 0.05 by two-tailed unpaired Student’s t-test **(B)**.

We calculated cuproptosis activity scores for atherosclerotic and normal vessel samples. Compared to normal vessels, cuproptosis scores were significantly elevated in atherosclerosis (Figures 3A,B). Among all cells, macrophage exhibited the highest cuproptosis scores, followed by mast cells and VSMCs, suggesting potential activation of cuproptosis-related pathways in these cells within atherosclerosis (Figure 3C; Supplementary Table S2). To further evaluate key factors influencing cuproptosis levels in atherosclerosis, we excluded normal tissue (Figure 3D) and divided samples into high and low cuproptosis groups based on median cuproptosis scores (Figure 3E). We compared the expression of 18 established cuproptosis-related genes across major cell types in atherosclerotic plaques. NLRP3 was predominantly expressed in macrophage, FDX1 was enriched in mast cells, while GLS showed universal expression across nearly all cell types (Figure 3F). Analysis of metabolic pathways indicated that glycolytic pathways were significantly enhanced in the high cuproptosis score group, whereas lipid metabolism was markedly elevated in the low cuproptosis score group (Figure 3G). In addition, several pathways closely related to mitochondrial metabolism, including pyruvate metabolism, the TCA cycle, oxidative phosphorylation, and sulfur metabolism, were also differentially enriched, suggesting that cuproptosis in atherosclerosis is accompanied by broader metabolic reprogramming. Notably, these pathways are mechanistically relevant to cuproptosis, as this form of cell death depends on mitochondrial lipoylated proteins, active TCA-cycle function, and destabilization of iron–sulfur cluster proteins. At the single-cell level, endothelial cells and fibroblasts exhibited relatively active energy metabolism pathways, while mast cells showed a more pronounced lipid metabolic profile. Macrophages displayed activation across multiple metabolic programs, including glycolysis, pyruvate metabolism, and mitochondrial energy pathways, which may partly explain their higher cuproptosis scores in atherosclerotic lesions (Figure 3H).

### 3.3 Machine learning identification of key cuproptosis-related genes

**FIGURE 4:**
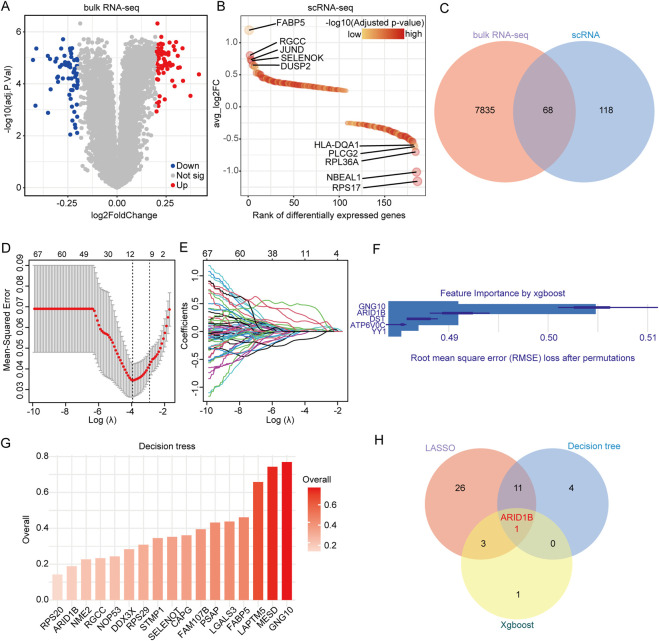
Machine learning Identification of the key cuproptosis-related gene. **(A)** Differentially expressed genes in high and low cuproptosis groups from bulk RNA-seq dataset GSE100927. **(B)** Differentially expressed genes in high and low cuproptosis groups from the scRNA-seq dataset. **(C)** Venn diagram displayed common differentially expressed genes. **(D)** The optimal penalty strength (λ) for the LASSO regression was selected via 10-fold cross-validation. **(E)** Coefficient distribution of key cuproptosis-related genes. **(F)** Importance ranking of key genes identified by XGBoost. **(G)** Decision tree modeling identifies key genes associated with cuproptosis-related genes. **(H)** Venn diagram showing genes shared by LASSO, XGBoost, and decision tree.

To identify key genes related to cuproptosis in atherosclerosis, we first screened differentially expressed genes between high and low cuproptosis score samples in both bulk RNA-seq and scRNA-seq datasets (Figures 4A,B). A total of 68 genes were commonly identified as differentially expressed across both datasets (Figure 4C). Subsequently, these 68 genes were individually incorporated into three types of machine learning algorithms, including LASSO cox regression, XGBoost, and decision trees. LASSO selected 41 candidate genes (Figures 4D,E; Supplementary Table S3), while XGBoost ranked the top five most important genes (Figure 4F; Supplementary Table S3). The decision tree identified 17 significant genes (Figure 4G; Supplementary Table S3). Integration of the results from all three algorithms pinpointed one gene: AT-Rich Interaction Domain 1B (ARID1B) (Figure 4H).

### 3.4 ARID1B affects the infiltration of immune cells in atherosclerosis

**FIGURE 5:**
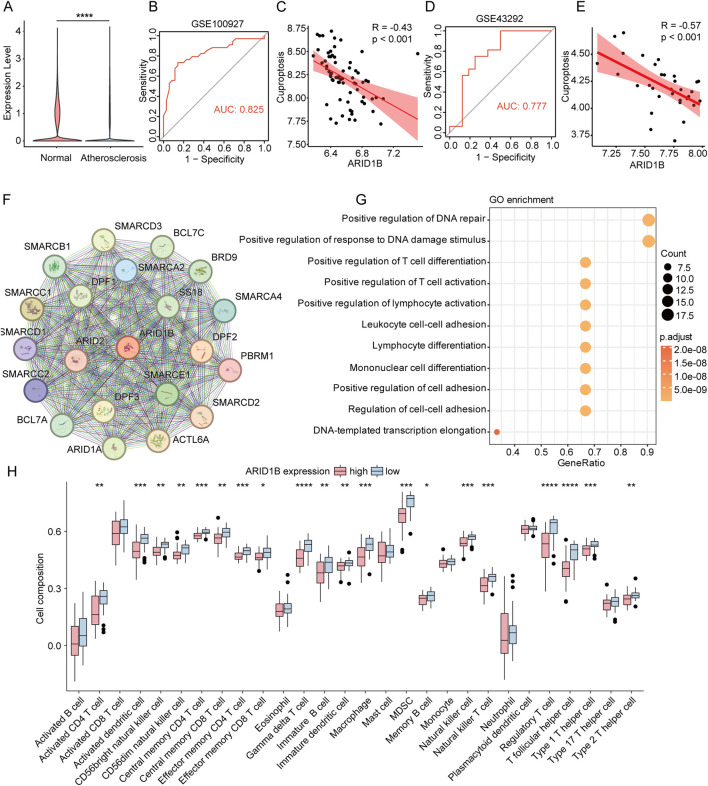
Function and immune infiltration analysis of ARID1B. **(A)** Expression of ARID1B in atherosclerosis and normal vessels. **(B)** Area under the curve for ARID1B diagnosis of cuproptosis. **(C)** Correlation curve between ARID1B expression and cuproptosis scores. **(D)** Validation of area under the curve for ARID1B diagnosis of cuproptosis in validation set. **(E)** Correlation curve between ARID1B expression and cuproptosis scores in validation set. **(F)** A PPI network centered on ARID1B. **(G)** GO Enrichment Analysis of the ARID1B PPI network. **(H)** Immune cell infiltration analysis in ARID1B high and low expression groups.

Compared to normal vessels, ARID1B expression were significantly reduced in atherosclerosis group (Figure 5A). Analysis of different cell types within cuproptosis subgroups revealed that ARID1B expression was downregulated in T cells and fibroblasts of the low cuproptosis group compared to the high cuproptosis group, whereas it was upregulated in other cell types (Supplementary Figure S1B). We also found that ARID1B could predict the level of cuproptosis, and the two showed a significant negative correlation (Figures 5B,C), which was also verified in another atherosclerosis dataset (GSE43292) (Figures 5D,E). We then constructed the PPI network of ARID1B (Figure 5F). The results of functional enrichment analysis indicated that the functions of the PPI network dominated by ARID1B mainly focused on the adhesion, activation, and differentiation of immune cells (Figure 5G). Given that the function of ARID1B may influence immune cell levels, we further analyzed the relative infiltration levels of immune cells in different atherosclerosis samples. The results indicated that there was more abundant infiltration of T cell and macrophage in the ARID1B low-expression group (Figure 5H). To clarify the interplay among ARID1B expression, cuproptosis activity, immune cell infiltration, and atherosclerotic plaque stage, we stratified samples according to ARID1B expression and cuproptosis scores, and then integrated immune cell infiltration levels with plaque classification. As illustrated by the Sankey plots (Supplementary Figure S1C–E), specimens with low ARID1B expression were predominantly mapped to higher cuproptosis scores and increased T cell and macrophage infiltration, and were more frequently assigned to advanced (type III–IV) lesions. In contrast, samples with high ARID1B expression tended to exhibit lower cuproptosis activity together with reduced T cell and macrophage infiltration, and were enriched in early-stage (type I–II) plaques. Collectively, these findings suggest that ARID1B may be involved in plaque progression by modulating cuproptosis-related processes and shaping immune cell recruitment within the lesion microenvironment.

### 3.5 The level of ARID1B affects intercellular interactions

**FIGURE 6:**
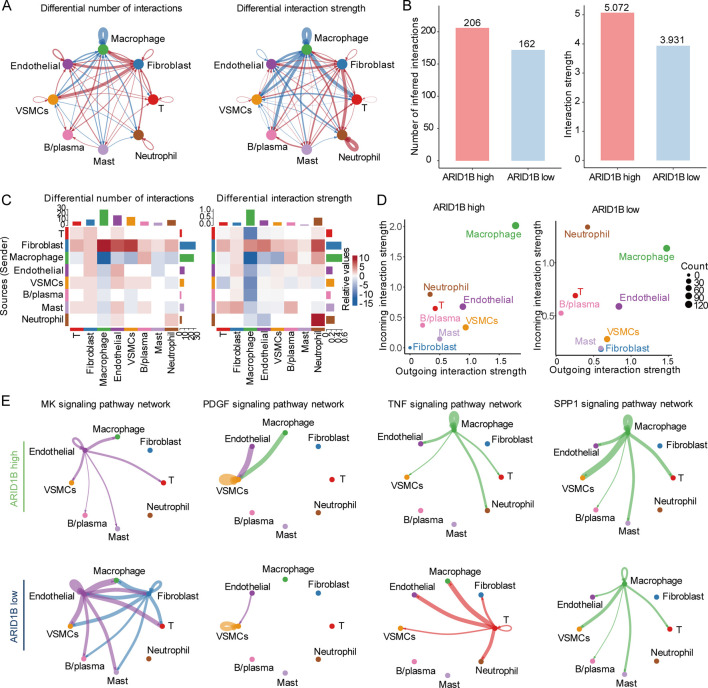
Differences in cell-cell interaction relationships between ARID1B high and low expression groups. The number **(A)** and strength **(B)** of intercellular interactions in ARID1B high and low expression groups. Red lines indicated stronger performance in the ARID1B high group, while blue lines indicate stronger performance in the ARID1B low group. **(C)** Heat maps illustrate the strength of interactions between different cells. **(D)** A group of cells in 2D space that undergo significant changes in outgoing or incoming signal. **(E)** Differences in cell interactions in key signaling pathways between ARID1B high and low groups.

To evaluate the potential impact of ARID1B on immune infiltration during atherosclerotic progression, we classified atherosclerosis samples into high and low ARID1B expression groups and analyzed differences in cell communication using CellChat. Specifically, compared to the ARID1B low-expression group, the ARID1B high-expression group exhibited stronger interactions between T cells and other cell types (red lines), while interactions between macrophage and other cells were weaker (blue lines) (Figure 6A). Furthermore, the ARID1B-high group demonstrated both a higher number and greater intensity of cell-cell interactions (Figure 6B). A heatmap detailed interaction strengths between different cell types (Figure 6C). A two-dimensional scatter plot visualized primary signal senders and targets, revealing macrophages as the predominant signal senders and receivers. Within the low ARID1B group, T cells and neutrophil exhibited stronger signal reception, while endothelial cells and VSMCs showed stronger signal transmission (Figure 6D). Regarding pathway-specific interactions, those promoting inflammatory cell infiltration and plaque formation showed stronger interactions in the ARID1B-low group. Conversely, SPP1, a key pathway suppressing atherosclerosis formation and promoting post-injury vascular repair, exhibited stronger activity in the ARID1B-high group (Figure 6E) (Isoda et al., 2002).

### 3.6 Experimental validation of optimal cuproptosis-related gene

**FIGURE 7:**
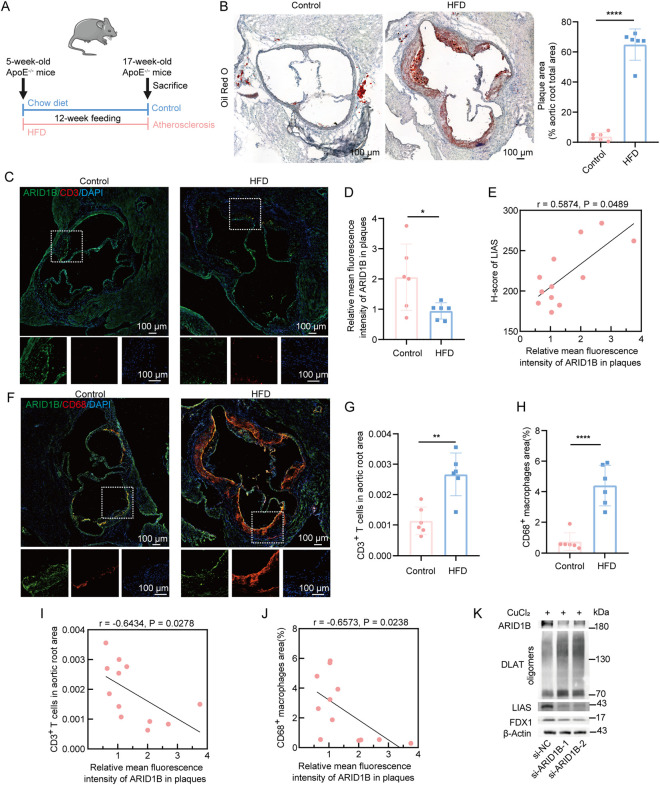
Cuproptosis-related gene ARID1B is correlated with macrophage and T cell infiltration during atherosclerotic progression. **(A)** Schematic of workflow for animal study. **(B)** Representative images and quantification of ORO staining of aortas from ApoE^−/−^ mice (*n* = 6). Scale bar: 100 µm. **(C)** Representative images of ARID1B (green) and CD3 (red) immunofluorescence staining in aortic root sections of the control and atherosclerosis groups, with nuclei counterstained with DAPI (blue). The area within the white dashed rectangle is magnified below. Scale bar: 100 µm. **(D)** The quantification of the relative mean fluorescence intensity of ARID1B in plaque area (*n* = 6). **(E)** The correlation analysis between H-score of LIAS and the relative mean fluorescence intensity of ARID1B (*n* = 12). **(F)** Representative images of ARID1B (green) and CD68 (red) immunofluorescence staining in aortic root sections of low and high cuproptosis group, with nuclei counterstained with DAPI (blue). The area within the white dashed rectangle is magnified below. Scale bar: 100 µm. The quantification of CD3^+^ T cells **(G)** and the percentage of CD68^+^ macrophages **(H)** infiltrating in the aortic root (*n* = 6). The correlation analysis between H-score of LIAS and the number of CD3^+^ T cells **(I)** and the percentage of CD68^+^ macrophages **(J)** (*n* = 12). **(K)** Western blotting analysis of cuproptosis-related proteins in THP-1 macrophages following ARID1B knockdown with 1 μM CuCl_2_ treatment. *P < 0.05, **P < 0.01, ***P < 0.001, ****P < 0.0001 by two-tailed unpaired Student’s t-test **(B,D,G,H)** or Spearman’s rank correlation coefficient **(E,I,J)**.

To investigate cuproptosis during atherosclerosis, ApoE^−/−^ mice received a 12-week HFD to induce atherosclerosis, with age-matched chow-fed mice as controls (Figure 7A). ORO staining of the aortic root sections confirmed the successful establishment of the atherosclerotic model (Figure 7B). Cuproptosis initiation is characterized by intracellular copper accumulation triggering decreased FDX1 expression, reduced LIAS levels, and increased DLAT oligomerization (Tsvetkov et al., 2022). The activation of cuproptosis within atherosclerotic aortic tissues was supported by the expression of core markers. Serum copper concentrations were significantly elevated in atherosclerosis groups versus controls (Supplementary Figure S2A). The suppression of FDX1 and LIAS expression alongside the pronounced oligomerization of DLAT was observed in the atherosclerotic group (Supplementary Figure S2B,C). Immunohistochemical staining revealed a decrease in LIAS expression, a specific target of cuproptosis (Tsvetkov et al., 2022), across atherosclerosis groups (Supplementary Figures S2D,E). The strong negative correlation (r = −0.7413) between LIAS expression and atherosclerotic plaque area supported the activation of cuproptosis in atherosclerosis group (Supplementary Figure S2F). Furthermore, a significant reduction of ARID1B expression in the atherosclerosis group was observed (Figures 7C,D). LIAS expression levels were significantly correlated with ARID1B expression (r = 0.5874) (Figure 7E). We verified the correlation of ARID1B on immune cell infiltration during atherosclerosis. CD3^+^ T cells count (Figures 7C,G) and CD68^+^ macrophages area (Figures 7F,H) were upregulated in the atherosclerosis group. Correlation analysis showed that ARID1B expression levels exhibited statistically significant negative correlations with CD3^+^ T cells count (r = −0.6434) (Figure 7I) and CD68^+^ macrophages area (r = −0.6573) (Figure 7J). To investigate the role of ARID1B in cuproptosis, we performed ARID1B knockdown in macrophages combined with copper treatment. This intervention significantly reduced FDX1 and LIAS protein expression while enhancing DLAT lipoylation, confirming activation of the cuproptosis pathway (Figure 7K). Collectively, these results demonstrated a potential correlation between cuproptosis-related gene ARID1B in modulating immune cell infiltration during atherosclerotic progression.

## 4 Discussion

Atherosclerosis, as a key driver of the global prevalence of cardiovascular disease, has become the leading cause of death and disability. Its central pathological process begins with endothelial cell injury, allowing low-density lipoprotein to infiltrate the vascular endothelium and trigger persistent inflammatory responses (Roth et al., 2020). Copper is critical for sustaining normal physiological processes across multiple biological systems. Epidemiological evidence confirms that copper intake (Eshak et al., 2018; Yang et al., 2022) and copper dyshomeostasis (Chen X. et al., 2023; Lin et al., 2024; Zou et al., 2025) have been implicated in various atherosclerosis-associated cardiovascular diseases, including peripheral arterial disease, stroke, and heart failure. Cuproptosis, a newly identified FDX1-regulated form of PCD characterized by lipoylated DLAT aggregation and LIAS depletion (Tsvetkov et al., 2022), is closely implicated in the development and progression of atherosclerosis (Chen et al., 2022). Previous studies suggest that targeting cuproptosis, such as using copper chelators like tetrathiomolybdate or modulating related genes, has emerged as a potential therapeutic strategy (Hu et al., 2022). Therefore, cuproptosis-associated genes hold promise as biomarkers for diagnosing atherosclerosis. Here, we integrated multiple scRNA-seq and bulk RNA-seq datasets to explore the characteristics of cuproptosis-related genes in atherosclerosis patients. ARID1B was identified as a cuproptosis-related gene inversely correlated with immune cell infiltration in atherosclerosis. Lower ARID1B levels were furthermore linked to more advanced atherosclerotic lesions. This significantly advances our understanding of cuproptosis-related immune dysregulation in atherosclerosis.

Atherosclerosis is fundamentally a metabolic inflammatory disease, whose onset and progression are accompanied by complex inflammatory and metabolic reprogramming. Intracellular metabolic reprogramming is induced by proatherogenic factors such as hypoxia, modified lipoproteins, and inflammatory cytokines (Groh et al., 2018). Mirroring metabolic adaptations often observed in tumor cells, vascular endothelial cells and monocytes undergo a shift toward glycolytic dependence and enhanced fatty acid synthesis (Groh et al., 2018; Luo et al., 2024). However, fundamental differences exist in mechanisms such as immune microenvironment and metabolic adaptation between atherosclerosis and cancer. Research revealed that glycolysis exhibits paradoxical roles in atherosclerosis development. While a metabolic shift from oxidative metabolism to glycolysis enhances adaptive survival under hypoxic and inflammatory stress (Xu et al., 2014; Yang et al., 2018), glycolytic reprogramming concurrently promotes plaque progression by accumulating intermediate metabolites that alter the vascular inflammatory microenvironment and exacerbate plaque progression (Liu et al., 2024; Roy et al., 2022). While glycolytic reprogramming in tumors has been shown to suppress cuproptosis (Song et al., 2025; Xing et al., 2023), whether cuproptosis influence metabolic alterations in atherosclerotic environments remains unclear. We found that compared to atherosclerosis plaque with a low cuproptosis score, the glycolytic pathway was significantly enhanced in the group with a high cuproptosis score. Developing targeted therapeutics for these cuproptosis-related metabolic pathways is expected to be an emerging therapeutic direction.

Using multiple machine learning algorithms, we identified ARID1B as a potential cuproptosis-associated biomarker associated with atherosclerotic pathology. The protein it encodes serves as a core subunit of the SWI/SNF chromatin remodelling complex. Through its ARID domain, it recognizes AT-rich regions in the genome and utilizes ATP hydrolysis to alter nucleosome positioning (Helming et al., 2014; Wang et al., 2020). Thus, it functions as a central epigenetic regulator, directly controlling chromatin accessibility to participate in the transcriptional activation and repression of downstream genes (Chen et al., 2024b). It modulates gene transcription by opening or closing chromatin structures and engages in extensive crosstalk with modification systems such as histone acetylation/methylation, collectively fine-tuning gene expression patterns. Multiple studies indicate significant methylation of ARID1B across various diseases (Kommoss et al., 2022; van der Sluijs et al., 2025). Consequently, utilizing epigenetic inhibitors (e.g., DNA methyltransferase inhibitors, histone acetyltransferase inhibitors) may represent a potential strategy to improve outcomes for atherosclerosis patients. Furthermore, prior studies have reported that ARID1B deficiency enhances DNA damage and activates the cGAS-STING pathway, inducing the release of type I interferons and numerous inflammatory cytokines (Watanabe et al., 2014; Zhu et al., 2024). In atherosclerosis, activation of cGAS-STING pathway may further amplify inflammation, leading to increased plaque instability (Li et al., 2023; Pham et al., 2021). We observed an increase in T cell and macrophage infiltration in atherosclerotic lesions, accompanied by a decrease in ARID1B expression. Based on previous articles, we propose the hypothesis that cGAS-STING represents a druggable target for ARID1B-hypofunction patients. Further investigation is required to delineate therapeutic effect and underlying mechanisms.

While this study offers novel insights, several limitations should be acknowledged. By analyzing multiple scRNA-seq and bulk RNA-seq datasets, we observed that ARID1B expression is reduced in groups with high cuproptosis scores and correlated inversely with immune infiltration levels, thus implicating its involvement in advancing atherosclerosis. However, the exact nature of this relationship and underlying mechanism remains unclear. Secondly, our study focused on RNA-level expression changes, though ARID1B mutations could also influence atherosclerosis. In addition, due to the lack of sex annotations in the available datasets, the role of sex in cuproptosis-related mechanisms in atherosclerosis could not be analyzed in the present study. Further clinical studies using atherosclerotic arterial samples are required to validate the therapeutic potential of targeting ARID1B. During cuprotosis, the iron-sulfur cluster proteins FDX1 and LIAS undergo progressive depletion. Given that iron-sulfur cluster biosynthesis requires sulfur metabolites as essential substrates (Maio et al., 2016; Tsaousis, 2019), combined with our GO enrichment analysis revealing significant upregulation of sulfur metabolism enrichment in the high cuproptosis score group, we hypothesizes that elevated cuproptosis activity may consequently trigger compensatory sulfur metabolism enrichment. The precise interplay between cuprotosis and sulfur metabolism requires further experimental validation.

In summary, ARID1B showing a correlation with plaque progression by modulating cuproptosis-related processes and shaping immune cell infiltration. This study offers a novel perspective on understanding the associations of cuproptosis and immune infiltration in atherosclerotic progression. Based on its association with increased infiltration of T cell and macrophage, we propose the cuproptosis-related gene ARID1B as a candidate biomarker for refining atherosclerosis treatment strategies.

## Data Availability

The original contributions presented in the study are included in the article/Supplementary Material, further inquiries can be directed to the corresponding authors.
