# Supplementary material for: Multi-omics integration identifies ARID1B linking cuproptosis-immune crosstalk with atherosclerotic plaque progression
Source: Front Genet. 2026 May 18;17:1795872. doi: 10.3389/fgene.2026.1795872 (PMC13222667; doi:10.3389/fgene.2026.1795872)
Supplement: Supplementary file 1 [file DataSheet1.docx]

Supplementary Material

# Supplementary Figure and Tables

- 1. **Supplementary Figures**


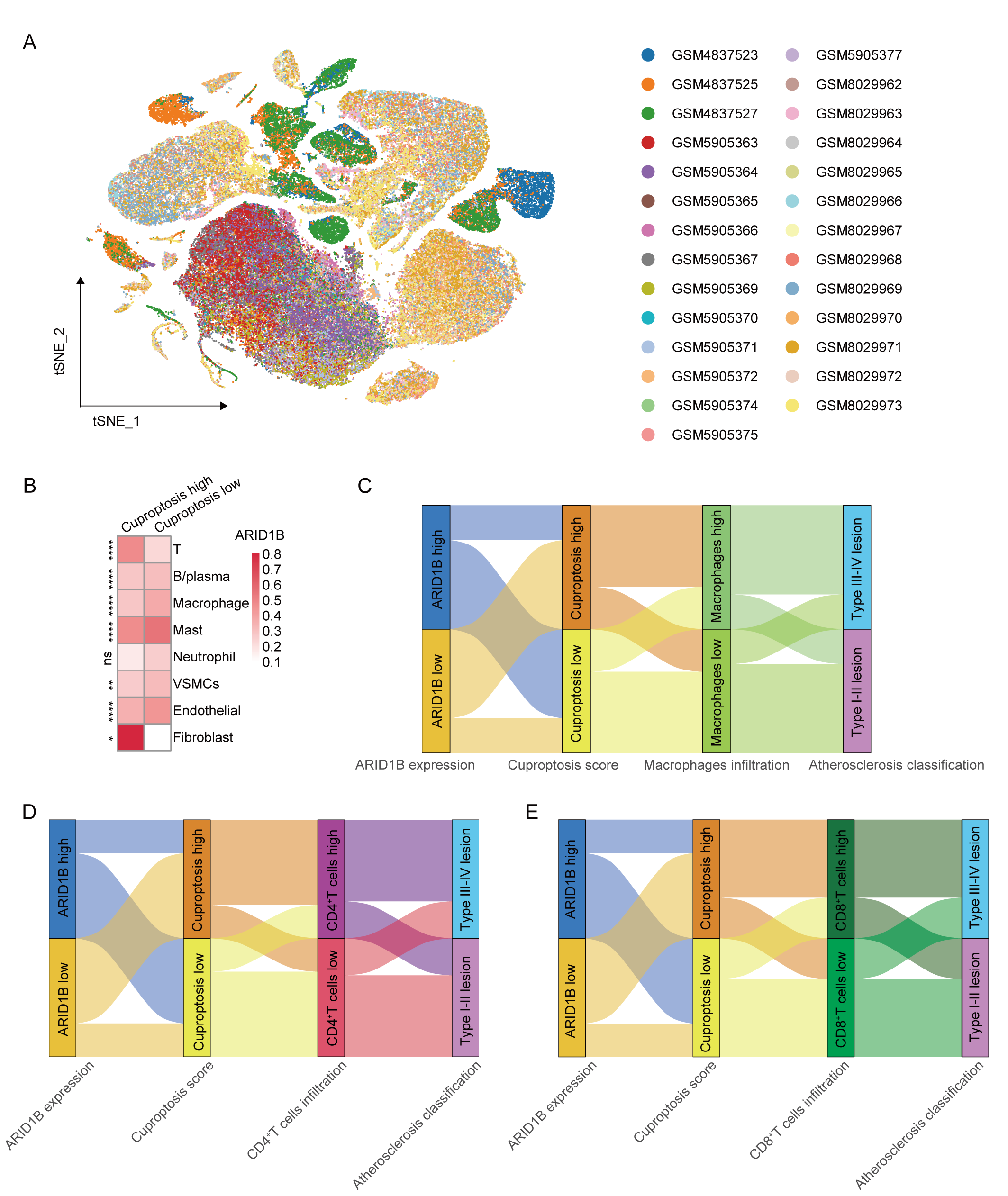


**Supplementary Figure S****1.** ARID1B expression and its pathogenic associations. **(A)** t-SNE visualization on all integrated samples. **(B)** Expression of ARID1B in different cells. Sankey diagram illustrating the relationship among ARID1B expression, cuproptosis activity, atherosclerotic plaque classification, and immune cell infiltration. The immune infiltration includes macrophages **(C)**, CD4^+^ T cells **(D)**, and CD8^+^ T cells **(E)**. Box heights correspond to relative proportions, while link widths indicate the relationship. ns = not significant, **P* < 0.05, ***P* < 0.01, ****P* < 0.001, *****P* < 0.0001.


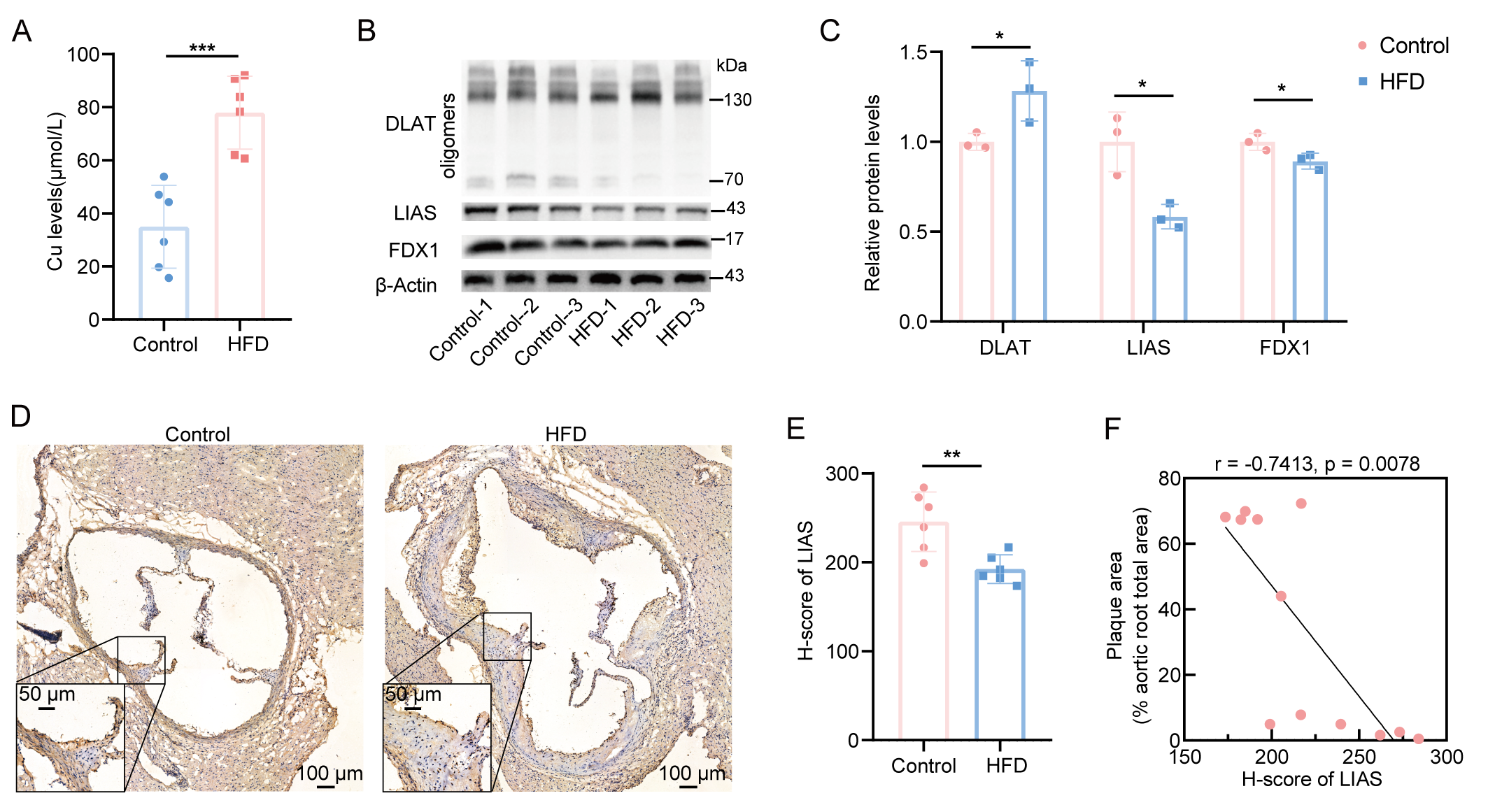


**Supplementary Figure S2.** Cuproptosis was activated in atherosclerosis. **(A)** Serum copper levels of ApoE^-/-^ mice were assessed via colorimetric assays (*n*=6). Representative Western blot images **(B)** and quantitative analysis **(C)** of cuproptosis-related proteins in aortic tissues. Representative images **(D)** and quantification **(E)** of immunohistochemical staining of LIAS. Scale bar: 100 µm. **(F)** The correlation analysis between H-score of LIAS and plaque area (*n*=12). **P* < 0.05, ***P* < 0.01, ****P* < 0.001 by two-tailed unpaired Student’s t-test (A, C and E) or Spearman’s rank correlation coefficient (F).

- 1. **Supplementary Tables**

**Supplementary Table S1.** List of 18 established cuproptosis-relatled genes.

| Cuproptosis-relatled genes | Full name | Gene ID | Function annotation | Reference |
| --- | --- | --- | --- | --- |
| FDX1 | Ferredoxin 1 | 2230 | Reductase; Cu^+^ chaperone; Member of iron-sulfur cluster protein; Induce lipoylated protein aggregation | Figure 3A and Supplementary Table S2 of Tsvetkov et al. 2022 |
| LIAS | Lipoic acid Synthetase | 11019 | Lipoyl moiety synthesis; Member of iron-sulfur cluster protein | Figure 3A and Supplementary Table S2 of Tsvetkov et al. 2022 |
| LIPT1 | Lipoyltransferase 1 | 51601 | Iron-sulfur assembly partner; Component of the lipoic acid pathway | Figure 3A and Supplementary Table S2 of Tsvetkov et al. 2022 |
| DLD | Dihydrolipoamide Dehydrogenase | 1738 | Component of the lipoic acid pathway; Component of PDH complex; Redox sensor | Figure 3A and Supplementary Table S2 of Tsvetkov et al. 2022 |
| DLAT | Dihydrolipoamide S-Acetyltransferase | 1737 | Component of PDH complex; Lipoylated TCA cycle protein; Copper-binding node | Figure 3A and Supplementary Table S2 of Tsvetkov et al. 2022 |
| PDHA1 | Pyruvate Dehydrogenase E1 Subunit Alpha 1 | 5160 | Component of PDH complex | Figure 3A and Supplementary Table S2 of Tsvetkov et al. 2022 |
| PDHB | Pyruvate Dehydrogenase E1 Subunit Beta | 5162 | Component of PDH complex | Figure 3A and Supplementary Table S2 of Tsvetkov et al. 2022 |
| MTF1 | Metal Regulatory Transcription Factor 1 | 4520 | Regulate metallothionein expression; Copper detoxifier | Figure 3A and Supplementary Table S2 of Tsvetkov et al. 2022 |
| GLS | Glutaminase | 2744 | Glutaminolysis regulator; Rescue TCA cycle collapse | Figure 3A and Supplementary Table S2 of Tsvetkov et al. 2022 |
| CDKN2A | Cyclin Dependent Kinase Inhibitor 2A | 1029 | Modulate cell cycle arrest | Figure 3A and Supplementary Table S2 of Tsvetkov et al. 2022 |
| LIPT2 | Lipoyltransferase 2 (Mitochondrial) | 387787 | Iron-sulfur assembly partner; Component of the lipoic acid pathway | Figure 3B and Supplementary Table S2 of Tsvetkov et al. 2022 |
| GCSH | Glycine Cleavage System Protein H | 2653 | Lipoylated TCA cycle protein; Iron-sulfur cluster biogenesis regulator | Figure 3B and Supplementary Table S2 of Tsvetkov et al. 2022 |
| DLST | Dihydrolipoamide S-Succinyltransferase | 1743 | Lipoylated TCA cycle protein; Component of the α-ketoglutarate dehydrogenase complex | Figure 3D and Supplementary Table S5 of Tsvetkov et al. 2022 |
| DBT | Dihydrolipoamide Branched Chain Transacylase E2 | 1629 | Lipoylated TCA cycle protein; Branched-chain ketoacid dehydrogenase complex subunit | Figure 3D and Supplementary Table S5 of Tsvetkov et al. 2022 |
| NLRP3 | NLR Family Pyrin Domain Containing 3 | 114548 | Inflammasome sensor activated by mitochondrial damage | Figure 1B and Supplementary Material 6 of Tang et al. 2024 |
| ATP7B | ATPase Copper Transporting Beta | 540 | Copper exporter | Figure 1B and Supplementary Material 6 of Tang et al. 2024 |
| ATP7A | ATPase Copper Transporting Alpha | 538 | Copper importer | Figure 1B and Supplementary Material 6 of Tang et al. 2024 |
| SCL31A1 | Solute Carrier Family 31 Member 1 | 1317 | High-affinity copper importer | Figure 1B and Supplementary Material 6 of Tang et al. 2024 |

**Supplementary Table S2.** The quantitative cuproptosis scores for major cell types of atherosclerotic and normal vessel samples.

| Cell type | Cuproptosis score |
| --- | --- |
| T cell | -0.031714526 |
| Endothelial | -0.052 |
| Fibroblast | -0.05 |
| Neutrophil | -0.036557027 |
| B/plasma | -0.031670352 |
| VSMCs | -0.030960307 |
| Mast cell | -0.030576667 |
| Macrophage | -0.026933499 |

**Supplementary Table S3.** List of differentially expressed genes related to cuproptosis in atherosclerosis identified as significant by LASSO cox regression, XGBoost, and decision trees.

| Machine learning algorithms | LASSO | Decision tree | Xgboost |
| --- | --- | --- | --- |
| Genes | LAPTM5 | ARID1B | GNG10 |
|  | FABP5 | CAPG | ARID1B |
|  | CAPG | DDX3X | DST |
|  | MESD | FABP5 | ATP6V0C |
|  | GNG10 | FAM107B | YY1 |
|  | SELENOS | GNG10 |  |
|  | ATP6V0C | LAPTM5 |  |
|  | SELENOW | LGALS3 |  |
|  | CSTB | MESD |  |
|  | PSAP | NME2 |  |
|  | LGALS3 | NOP53 |  |
|  | FTH1 | PSAP |  |
|  | JPT1 | RGCC |  |
|  | NSD3 | RPS20 |  |
|  | YY1 | RPS29 |  |
|  | PSMA6 | SELENOT |  |
|  | HLADRA | STMP1 |  |
|  | B2M |  |  |
|  | FYB1 |  |  |
|  | ARID1B |  |  |
|  | BRD7 |  |  |
|  | SELENOT |  |  |
|  | CCL4 |  |  |
|  | RGS1 |  |  |
|  | ATP5PB |  |  |
|  | CTSL |  |  |
|  | MCUB |  |  |
|  | HSPA1B |  |  |
|  | RPS29 |  |  |
|  | RGCC |  |  |
|  | HIST1H4C |  |  |
|  | RPL17 |  |  |
|  | FAM177A1 |  |  |
|  | ARPC1B |  |  |
|  | DDX3X |  |  |
|  | NAP1L1 |  |  |
|  | NOP53 |  |  |
|  | MIF |  |  |
|  | NBEAL1 |  |  |
|  | ATP5MC3 |  |  |
|  | RPL4 |  |  |
